# Outcome analysis of differentiated thyroid cancer: Experience from tertiary care in Karachi

**DOI:** 10.12669/pjms.40.11.7036

**Published:** 2024-12

**Authors:** Saira Furqan, Asma Ahmed, Sumera Batool, Najmul Islam

**Affiliations:** 1 Saira Furqan, MBBS, FCPS (Medicine), FCPS (Endocrinology) Assistant Professor, Endocrinology Diabetes and Metabolism, Department of Medicine, The Aga Khan University Hospital, Karachi, Pakistan; 2 Asma Ahmed, MBBS, MRCP, Certificate in Diabetes & Endocrinology Assistant Professor, Endocrinology Diabetes and Metabolism, Department of Medicine, The Aga Khan University Hospital, Karachi, Pakistan; 3 Sumera Batool, MBBS, FCPS (Medicine), FCPS (Endocrinology) Consultant, Endocrinology Diabetes and Metabolism, Department of Medicine, The Aga Khan University Hospital, Karachi, Pakistan; 4 Najmul Islam, MBBS, MRCP, FRCP. Professor, Endocrinology Diabetes and Metabolism, Department of Medicine, The Aga Khan University Hospital, Karachi, Pakistan

**Keywords:** Differentiated thyroid cancer, Papillary carcinoma, Follicular carcinoma, Hurthle cell variant of follicular carcinoma

## Abstract

**Objective:**

Although differentiated thyroid cancers have a good prognosis overall, their incidence is on the rise with extremely limited data available in our region. The objective was to describe the outcomes of differentiated thyroid carcinoma in a tertiary care hospital.

**Methods:**

This was a retrospective study conducted at Aga Khan University Hospital. The data was collected by reviewing the charts and reports of patients (1999-2011) diagnosed with differentiated thyroid cancer. Information regarding the demographic status, type of differentiated thyroid cancer, stage at the time of presentation, and the outcome was noted. The study endpoints were the disease cure, persistence, and recurrence.

**Results:**

Most of the patients were diagnosed with papillary carcinoma stage-I (49.0%) having a cure rate of 52.94%, a persistence rate of 28.43%, and a recurrence rate of 18.62%. A 33.3% of patients with follicular carcinoma at stage-4 had zero cure rate, the persistence rate was 25% and the recurrence rate was also 25%. In patients with hurthle cell variant of follicular carcinoma; the majority (60.0%) were diagnosed at stage-I having a cure rate of 80%. The persistence rate was 0%, and the recurrence rate was 20%.

**Conclusion:**

This study highlights the low cure rate while describing the high rate of disease persistence and recurrence. This can be due to delays related to the diagnosis and scarcity of proper treatment. Recognition of this emergent disease is vital so that curative methods prescribed at proper time can diminish the morbidity and mortality linked with this treatable cancer.

**List of Abbreviations:**
(WDTC): Well-Differentiated Thyroid Cancer,
(TC): Thyroid Cancer/,
(RAI): Radioactive Iodine Ablative Therapy,
(CT scan): Computerized Tomography Scan,
(OS): Overall Survival,
(AJCC): American Joint Committee on Cancer,
(FTC): Follicular Thyroid Carcinoma,
(PTC): Papillary Thyroid Carcinoma,
(NCDB): National Cancer Database,
(HCC): Hurthle Cell Carcinoma.

## INTRODUCTION

It is a profound concept in the healthcare field that differentiated thyroid carcinoma (DTC) has a good survival rate when compared to other malignancies.^1^,^2^ However, it is also reported that there is an increase in the DTC rate with a representation of 3% percent in the worldwide incidence rate of all malignancies.^3^ DTC is estimated to be the 4th commonest cancer in 2030,^4^,^5^ despite the convenience of prior diagnosis and availability of better treatment options.^6^

The increase in the prevalence rate of TC can be because of the upsurge in awareness regarding this disease and the correct utilization of diagnostic modalities within the preclinical stages.^4^,^6^,^7^ Due to the increasing number of cases, there is a dire need to collect and understand the correct data relating to factors and predictions of DTC. In Pakistan, the incidence of DTC is 1.2% among all malignant tumors.^5^ This rise in the cases is irrespective of gender and is reported to be increased from 3.9 to 23.4/100,000 in females and from 1.5 to 7.2/100,000 in males since 1970 with no significant change in mortality.^1^

Various studies have been conducted to evaluate the predictive factors relating to DTC.^8^,^9^ However, it is being investigated as to whether these factors are to be applied with equal effectiveness to the patients who have different racial and geographical backgrounds. The prognosis of DTC is largely dependent on the TNM stage and histological type.^10^,^11^ Our research aimed to evaluate the TNM stage of DTC at a time point of identification of the disease in our population and substantial to predict the outcome.

## METHODS

This study was carried out in the Aga Khan University Hospital Karachi (AKUH), which is a tertiary care teaching facility having a capacity to cater to 560 inpatients. The cases of DTC have been selected by a computerized search with the help of the hospital information management system (HIMS department). Around the year 1999 to 2011, 206 patients presented to the hospital and were appropriate to our inclusion criteria. Out of these 206 patients, 119 were chosen for the retrospective analysis. These patients were monitored for five years for the results in terms of cure, recurrence, or persistent disease.

Data concerning the demographic status, type of DTC, stage of cancer at the time of diagnosis, and the effect of the treatment was analyzed. The outcomes of prognosis were derived from the follow-up information presented in the files. Consequently, the duration of follow-ups was also calculated from the time of the last evaluation. The endpoints of this research were the cure, persistence, and recurrence.

Recurrence is described as the re-occurrence of the cancer traces in the patient who has undergone a curative surgery (Total thyroidectomy with lymph node dissection) followed by successful implication of radioactive iodine ablative therapy (RAI). In our study, the recurrence was identified through the imaging modalities such as ultrasound, CT scan, whole-body scan, and serum thyroglobulin level monitoring. Recurrence can be localized, arising in the bed of the thyroid or cervical lymph nodes and it can be distant including metastasis to other organs like lungs or bones.

Persistence is described as the cases that were not cured after undergoing surgical and RAI therapy. These patients had persistently increased thyroglobulin levels even in the absenteeism from the intrusion of anti-thyroglobulin antibodies with or without radiological evidence of diseases persistence linked with this disease.

Cure is the non-existence of local and or distant disease with normal stimulated thyroglobulin levels and normal imaging modalities following the initial procession of the curative surgery and RAI treatment.

### Ethical Approval:

An exemption of ethical review was taken from the Ethical Review Committee of AKUH (2246Med-ERC-12, dated June 22, 2012). The confidentiality of every participant was maintained by following the ethical code of conduct.

The names of the potential patients were not requested when the data was collected while only medical record numbers (MR Numbers) were requested. It was ensured that there was no misuse of the data. The finalized data was only accessible to the research team. Hence the data was stored in an online, password-protected database. Unique identifier numbers (UIN) were allotted to each participant to remove the MR numbers. It is also informed that this study has no associated risks or costs to the participants.

### Statistical Analysis:

In this study, the continuous variables were studied as mean and Standard Deviation (±SD), whereas the categorical variables were written as a percentage. The normality of the data was determined through a Kolmogorov-Smirnov test. The frequencies and percentages of variables such as stages cure rate, persistent rate, and recurrence rate were presented. Unpaired T-Test was applied to compare the continuous variables between groups whereas the categorical variables were evaluated by using the chi-square test for which a p-value of <0.05 was taken as significant. The data was statistically analyzed using the SPSS version 16.0.

## RESULTS

The incidence of DTC is increasing worldwide. In our study, 206 patients were diagnosed with DTC. Total of 87 participants have been excluded from the study because of the unavailability of complete data from the medical records. After which 119 participants were selected for the study. It was reported that the most prevalent type of TC in our study was Papillary Thyroid Carcinoma (PTC) (n=102) followed by Follicular Thyroid Carcinoma (FTC) (n=12) and the Hurthle Cell Carcinoma (HCC) variant of FTC (n=5).

The mean age reported in the study was 42±17 years. A female majority (66.4%) was observed in all subtypes of DTC with only 33.6% male representation (2:1 ratio). The participants were monitored for five years. Most of the participants with PTC (n=50, 49%) had stage-I diseases, whereas four (33.3%) patients with FTC were diagnosed at stage-IV. Though there were only five patients with HCC, 3(60.0%) who were diagnosed at stage-I. Further details in Chart-I.

**Chart-I:**
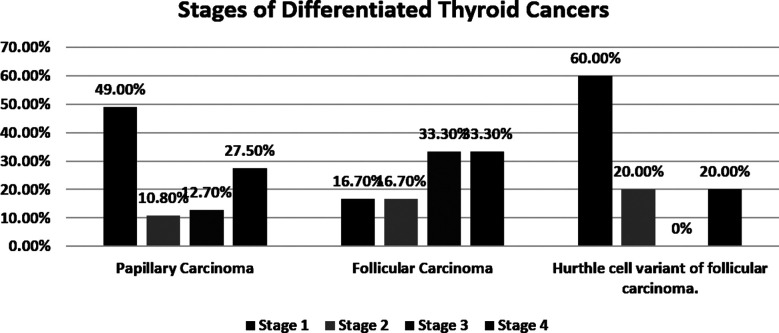
Stages of Differentiated Thyroid Cancers.

It is also important to note that the HCC had the highest cure rate which was 80% along with a zero-persistence rate and had only 20% of recurrence rate, which is the minimalist of all the three subtypes observed in our study. Further details in Chart-II.

**Chart-II:**
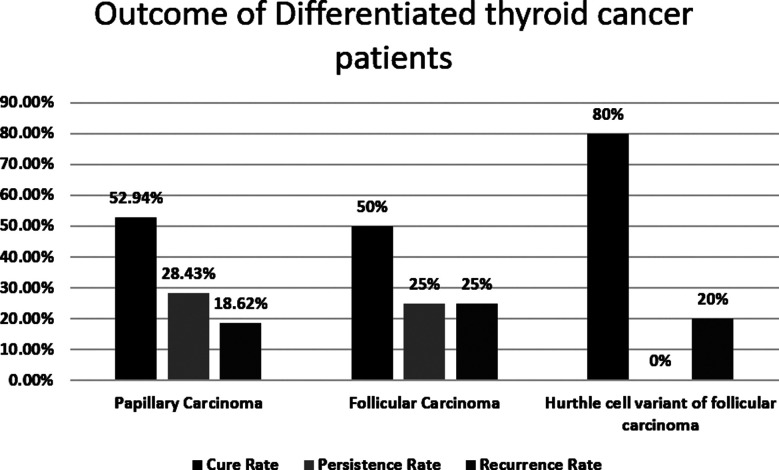
Outcome of Differentiated thyroid cancer patients.

## DISCUSSION

We reviewed 119 patients with WDTC who were surgically intervened for total thyroidectomy along with neck dissection. After the surgery, RAI treatment was given. The mean age of the patients in our study was 42 ± 17 years which can be assessed across the global as well as local data.^12^ Among DTC, FTC and HCC revealed a late mean age of 49 and 47 years respectively, when compared to the PTC which had a mean age of 42 years. According to the global population data, it is widely known that thyroid cancer has a high prevalence in female patients which is close to the 2:1 as presented in our study.^12^-^14^

During the previous decade, small-scale and single-country investigations and research have been published on WDTC. These papers have presented the incidence and also the presentation of this aggressive malignancy but, large-scale, multinational epidemiological studies are limited.^15^ Despite the increasing rate of this menacing disease, a massive number of patients are observed to experience long-term survival. It is also reported that a 10 years overall survival (OS) rate is observed at >90% after total or near-total thyroidectomy combined with radioiodine (131I) ablation therapy.^16^

It is apparent from both local and international literature that PTC is the most common variant of DTC followed by FTC.^17^,^18^ This is like the results of our study. There are various classifications for improving the risk-related assignment of individuals diagnosed with TC. The TNM classification is valid and reliable in determining the prognosis of TC. The TNM classification is further divided based on certain clinical and biological factors. The long-term outcome of TC is dependent upon the histological findings such as type of cancer, age, and the stage of carcinoma at the time of presentation.^19^

A research study conducted by Orosco RK et al. describes that more than 76% of patients with DTC presented at the initial stages whereas in our study it is reported that PTC (n=49%), FTC (n=16.7%), and patients of HCC variant (n=60%) were diagnosed at stage-I.^20^ Similarly, a study conducted in Canada comprised of patients from the CANNECT registry (2000 to 2010), showed that 69.2% (n=2529) of patients were diagnosed with the American Joint Committee on Cancer (AJCC) stage-I.^21^ A probable reason for the late presentation of disease in our population can be because of the lack of awareness, unavailability of suitable healthcare facilities, and failure to gain better healthcare services due to financial constraints.

According to studies it is reported that PTC is a popular and least antagonistic type of thyroid cancer because of its ability to increase in size and metastasize at a very low rate.^22^ In our study, we found that 59.8% were diagnosed with PTC at the initial stages (I and II) which is comparable to another study that showed the same results from the year 2004-2013. Many patients were diagnosed with stage I during these years with a mean rate of 8.34 patients out of 8.91 total thyroid cancer patients.^23^ However, a retrospective research study conducted through National Cancer Database (NCDB) showed that PTC represented 49.4% of stage IV cases^24^ so overall the results are quite variable.

FTC is the second most frequently diagnosed thyroid malignancy. It is more aggressive and metastasizes early as compared to PTC.^25^ A total of 33 patients in our study, who were diagnosed with FTC presented at stages-I and 2. A study conducted in Europe concluded that 75 out of 98 patients, i.e. 76.5% of patients with FTC were diagnosed at stage-I or II.^26^ So, the diagnosis of FTC at the preliminary stages has a rising incidence throughout the world. However, in our study, it was evident that FTC is mostly diagnosed at later stages largely because of the delay in diagnosis. In our study, the patients of FTC were presented at a late stage in comparison to PTC, the reason for which is unclear. However, it could be because of lack of awareness in our population to get evaluation for thyroid nodules or goiter at an early stage.

Intriguingly, the HCC is a variant of FTC which is contemplated as a more aggressive type of cancer than its correlated subtypes. It is associated with a higher mortality rate when compared to FTC.^27^ In our study, it is presented at an initial stage with 80% of patients presenting at stages-I and II. Moreover, there is zero persistence documented in our research, which contrasts with the reporting of other studies. Chindris et al report that between the years 2001 to 2012, out of 173 patients 60% male participants were diagnosed with widely invasive HCC variant (Stage-III and IV). It was also a risk factor for recurrence and mortality amongst the patients.^28^ With the diagnosis of HCC of FTC in early stages with zero persistence in our study, we are still not able to say that this variant is a less aggressive in the population because of a very small number of patients with this variant.

In patients with PTC, the persistence rate is 28.43%. These patients were diagnosed at a late stage. If the diagnosis of this tumor is done at the initial stage, the cure rate of this malignancy can be increased. A study conducted in Catania Italy reports that the patients with T1a (1.4%) and T1b (3.8%) had minimal chances of persistence. Similar was the data reported for the recurrence where low-risk patients (T1a and T1b) had a 0.9% recurrence rate. About 20% of the patients diagnosed with PTC had recurrence during an average follow up of 6 months to 10 years.^29^ This is comparable to our study which shows a recurrence rate of 18.62%. In our patients with FTC, the cure rate is 50%. However, another study showed that the cure rate in their patients was 10.1%.^30^

International data showed that the recurrence rate of the FTC variant is relatively high at 43.5%, and more than half of the recurrences were reported within three years.^31^ In our study, the recurrence is 25% while another study shows a recurrence rate of 15.6%. There are different schools of thought concerning the clinical behavior and outcome of the HCC variant of FTC. It is also reported in research studies that the HCC variant of FTC is more aggressive when compared to FTC and is also related to a higher mortality rate. Our study demonstrated a positive outcome with a cure rate of 80% but the recurrence rate, on the other hand, was 20% which is high in comparison to a large clinical study conducted by Chindris AM et al. The study shows patients with minimally invasive HCC had zero recurrences.^28^ However, our study had a major limitation to draw any conclusion regarding the HCC variant of FTC because of the small sample size.

It is a dilemma of our age that even after the availability of innovative molecular therapeutic approaches, the cure rate of DTC is still low, with high persistence and recurrence rates all because of delay in diagnosis and lack of proper treatment. According to the studies, this is a slow-growing malignancy, and it possess no danger to the quality of life up until the later stages. This is evident that the early diagnosis of this conceivably curable malignancy is the key to lowering the mortality rates. The early diagnosis involves a high index of suspicion with proper investigations and timely treatment.

### Limitations:

As this is a retrospective study and we reviewed previous patients’ charts and there were many patients with lost follow-up data. Hence, a prospective study with a longer duration is essential as this is a slow-growing malignancy.

## CONCLUSION

Our study demonstrated that in our population, differentiated thyroid cancer patients were presented at advanced stage as compared to international data, and there was a low cure rate amongst our population with a high rate of persistence and recurrence. This is because of the delay in diagnosis and lack of proper treatment. There are multiple reasons for this which include lack of awareness in the general population, lack of good healthcare facilities for early diagnosis and management of DTC and financial constraints to avail good healthcare. We recommend that there should be proper awareness campaigns against this growing malignancy like the breast, lung, and various other malignancies. It will be a crucial step for the curative measures taken to reduce the rates of these diseases.

### Authors’ Contribution:

**SF:** Initiated the research, wrote and corrected the manuscript.

**AA:** Provided her expertise in manuscript writing and correction, literature search, critical review.

**NI** and **SB:** Supervised this study along with providing their expertise in manuscript writing critical review. All authors have read the final manuscript and are also responsible for the accuracy or integrity of the work.
